# A novel lung-avoidance planning strategy based on 4DCT ventilation imaging and CT density characteristics for stage III non-small-cell lung cancer patients

**DOI:** 10.1007/s00066-021-01821-1

**Published:** 2021-08-05

**Authors:** AiHui Feng, Yan Shao, Hao Wang, Hua Chen, HengLe Gu, YanHua Duan, WuTian Gan, ZhiYong Xu

**Affiliations:** Department of Radiation Oncology, Shanghai Chest Hospital, Shanghai Jiao Tong University, NO.241 West Huaihai Road, Xuhui District 20030 Shanghai, China

**Keywords:** Pulmonary function, Functional imaging, HU value, Lung cancer, Deformable image registration

## Abstract

**Background:**

Functional planning based merely on 4DCT ventilation imaging has limitations. In this study, we proposed a radiotherapy planning strategy based on 4DCT ventilation imaging and CT density characteristics.

**Materials and methods:**

For 20 stage III non-small-cell lung cancer (NSCLC) patients, clinical plans and lung-avoidance plans were generated. Through deformable image registration (DIR) and quantitative image analysis, a 4DCT ventilation map was calculated. High-, medium-, and low-ventilation regions of the lung were defined based on the ventilation value. In addition, the total lung was also divided into high-, medium-, and low-density areas according to the HU threshold. The lung-avoidance plan aimed to reduce the dose to functional and high-density lungs while meeting standard target and critical structure constraints. Standard and dose–function metrics were compared between the clinical and lung-avoidance plans.

**Results:**

Lung avoidance plans led to significant reductions in high-function and high-density lung doses, without significantly increasing other organ at risk (OAR) doses, but at the expense of a significantly degraded homogeneity index (HI) and conformity index (CI; *p* < 0.05) of the planning target volume (PTV) and a slight increase in monitor units (MU) as well as in the number of segments (*p* > 0.05). Compared with the clinical plan, the mean lung dose (MLD) in the high-function and high-density areas was reduced by 0.59 Gy and 0.57 Gy, respectively.

**Conclusion:**

A lung-avoidance plan based on 4DCT ventilation imaging and CT density characteristics is feasible and implementable, with potential clinical benefits. Clinical trials will be crucial to show the clinical relevance of this lung-avoidance planning strategy.

**Supplementary Information:**

The online version of this article (10.1007/s00066-021-01821-1) contains supplementary material, which is available to authorized users.

## Introduction

Radiation pneumonitis (RP) usually occurs 1–6 months after radiotherapy and is one of the most common complications in patients with thoracic diseases. According to statistics, the incidence of ≥ grade 2 RP in lung cancer patients is 28% [1], while the incidence of fatal pneumonia is approximately 2% [2]. The risk of pneumonia is considered to be the main limiting factor of the prescription dose that can be safely delivered to lung cancer with a high risk of local failure.

There are plenty of reports about the predictors of RP. Among them, dose metrics, such as the percentage of lung volume receiving ≥ 20 Gy (V20), have been studied most intensively. However, the establishment of these predictive models is based on the assumption that the lungs are homogeneous and healthy. However, a number of studies have shown that there is substantial functional and structural heterogeneity within the lung that could be further exacerbated with the presence of disease, which is one of the reasons why these models cannot be used in clinical practice. Studies have demonstrated that functional dosimetric parameters yield a better indication of the incidence of RP [3–5] when compared to anatomic dosimetric parameters, such as pV20Gy_high (the cumulative lung volume that received more than 20 Gy in the high-perfusion region was obtained and normalized to the total healthy lung volume).

CT ventilation is a developing imaging method using four-dimensional computed tomography (4DCT) and deformation image registration. Through years of clinical verifications, 4DCT has been proven to provide ventilation images with reasonable correlation to nuclear medicine [6], hyperpolarized ^3^He MRI [7], xenon-CT [8], and pulmonary function tests [9]. The accuracy and reproducibility of CT ventilation imaging have been confirmed by animal studies [10] and human studies [11]. Compared with other functional imaging modalities, CT ventilation imaging requires no radioactive contrasts and has higher resolution, lower cost, shorter scanning time, and greater availability (it can provide anatomical information and ventilation function information at the same time). 4DCT is currently used routinely in many centers, and the calculation of ventilation images involves only image processing. Previous studies have confirmed the dosimetric [12] and biological benefits [13] of imaging-based functional-lung-avoidance plans.

A major limitation of imaging-based functional-lung-avoidance plans is that functional irreversibility of the lung is assumed. However, in the radiotherapy treatment process, tumor shrinkage and reperfusion of the atelectasis region will lead to changes in the functional area. Another alternative planning strategy to reduce lung toxicity is to avoid dose deposition in healthy lung areas that are highly sensitive to radiation damage. Preclinical studies have demonstrated that changes in lung density are closely related to histopathological radiation injury and physical endpoints [14]. The ΔHU value (defined as ΔHU = HU_follow-up_ – HU_0_) is considered a substitute for lung injury, and a strong correlation between ΔHU and baseline CT density has been established [15]. According to previous findings, a density increase of more than 20 HU is not to be expected in emphysematous areas but only in healthy lung tissue. Therefore, protecting the lung area with high HU can prevent irreparable damage in the healthy lung area to a certain extent. Defraene [16] confirmed the feasibility of planning design based on CT density to reduce lung toxicity.

Functional avoidance planning based on 4DCT ventilation imaging and CT density are two approaches to lower lung toxicity. In this study, we combined the above two characteristics to design a novel lung-avoidance plan, attempting to prevent radiation damage to lung portions with high function or high susceptibility, to reduce lung toxicity as much as possible. The coincidence coefficient between high-function areas and high-density areas was discussed. In addition, the novel lung-avoidance plan was compared with the anatomy-based clinical plan. This study aimed to develop a brand-new, convenient, simple, and implemental planning strategy to preserve functional lungs and healthy lungs by reducing radiation damage to the lung while improving the clinical benefits for patients with lung cancer.

## Materials and methods

### Patient dataset

Twenty lung cancer patients who received conventional intensity-modulated radiation therapy (IMRT) in Shanghai Chest Hospital were retrospectively selected. Patients received a median prescription dose of 57.9 Gy (range 50–60 Gy) in 29 fractions (range: 25–30 fractions). All patients underwent routine pretreatment 4DCT simulations. Table 1 lists the characteristics of patients.

**Table 1:** Patient characteristics (absolute number of patients or median value with range)

Characteristics	Number (%) or median (range)
*Number of patients*	20
*Age (years)*	64 (27–89)
*Gender*	
Male	12 (60)
Female	8 (40)
*COPD*	
None	16 (80)
Stage I	3 (15)
Stage II	1 (5)
*Interstitial lung diseases*	
Yes	1 (5)
No	19 (95)
*Atelectasis*	
Yes	3 (15)
No	17 (85)
*Emphysema*	
Yes	6 (30)
No	14 (70)
*Pack years*	17 (0–45)
*Stage*	
IIIA	7 (35)
IIIB	13 (65)
*PTV (cm*^*3*^*)*	301.98 (117.59–709.38)
*Lung minus GTV volume (cm*^*3*^*)*	3364.36 (2560.03–5066.24)

*COPD* chronic obstructive pulmonary disease, *PTV* planning target volume, *GTV* gross tumor volume

### CT ventilation image calculation

CT ventilation imaging is based on 4DCT deformation image registration and quantitative image analysis. SOMATOM Definition AS (Siemens Healthcare GmbH, Erlangen) was used to obtain the 4DCT scans. The following standard 4DCT scan parameters were used: 120 kVp, 3‑mm slices, and variable mAs. For reconstruction, the CT images were sorted into nine respiratory bins by the phase-based method using a Real-time Position Respiratory Gating System (version 1.7.5, Varian Medical Systems, Palo Alto, CA). Although respiratory guidance (such as audiovisual biofeedback) was not used in this study, we provided oral guidance for patients to maintain free breathing. In all CT scans, patients were positioned with the same specific fixation device to reduce setup variability. Each patient’s simulation 4DCT data were used to calculate 4DCT ventilation maps. The lungs were segmented on the end-inhale and end-exhale phases. Lung voxel elements were then mapped from the inhale to exhale phase using a DIR algorithm in MIM (MIM Maestro, version 7.0.4, MIM Software Inc, Cleveland, OH).

**Fig. 1:**
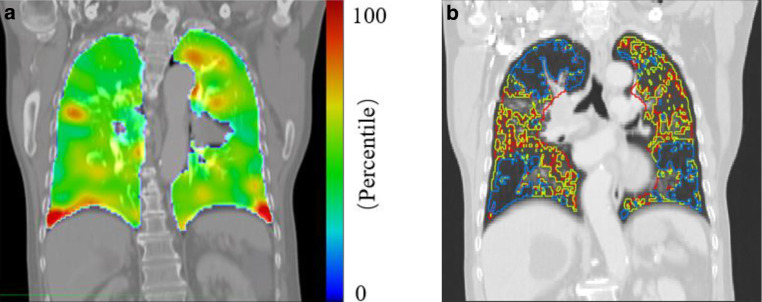
**a** Percentile ventilation maps for example cases. **b** Density maps for example cases

The three-dimensional deformation on the voxel base was recorded, and the Jacobian determinant was calculated based on Eq. 1 by using the MIM toolbar. A Jacobian determinant of 1 represents no volume change during the breathing motion. Voxelized ventilation was defined as a Jacobian determinant minus 1 (Eq. 2) with the assumption that ventilation is indicated by the power of volume changes in the voxels. The ventilation map is shown in Fig. 1a.

1 $$J ( x, y, z ) = \left \lfloor \begin{array}{c} 1 + \frac{du_{x} (x,y,z)}{dx}\ \frac{du_{x} (x,y,z )}{dy} \frac{du_{x}(x,y,z)}{dz}\\ \frac{du_{y}(x,y,z)}{dx}\ 1+\frac{du_{y}(x,y,z)}{dy} \frac{du_{y}(x,y,z)}{dz}\\ \frac{du_{z}(x,y,z)}{dx}\ \frac{du_{z}(x,y,z)}{dy} 1+\frac{du_{z} (x,y,z)}{dz} \end{array}\right\rfloor$$

2 $$\Updelta V=J\left(x,y,z\right)-1$$

### Defining anatomic, high-function, and high-density lung contours

Based on the voxelized ventilation values, the lung was separated into high-, medium-, and low-ventilation zones using the following thresholds: the highest 60% of the total voxels were deemed the high-ventilation region (H-V). The highest 30% of the total voxel minus the high-ventilation region was the medium-ventilation region (M-V). A rest area was defined as a low-ventilation region (L-V).

In addition, the total lung volume structure was divided into three separate “lung minus GTV” substructures combing lung voxels (the lung was first cropped by 5 mm to eliminate boundary effects) with similar CT densities: > −700 HU is considered a high-density area (H-D), −850 HU to −701 HU is a medium-density area (M-D), and < −850 HU is a low-density area (L-D). Separate 3D islands smaller than 1 cc were excluded from the structures. Contouring of the high-function and high-density lung portions was performed manually in MIM. The definition of the three lung substructures is outlined in Fig. 1b.

### IMRT optimization for ventilation and density-based lung-avoidance treatment planning

For each patient, two plans were generated: a lung-avoidance plan and a clinical plan. The design of lung-avoidance radiotherapy planning is to avoid lung portions with high function and high density, maintain target coverage, and meet the standard limits for critical organs (the standard constraints for planning target PTV and organ at risk [OAR] are listed in Supplementary Appendix Table A1).

The plans were created on Pinnacle^3TM^ treatment planning system (TPS, v9.10, Philips Medical Systems, Cleveland, USA) with the auto-planning module. All OARs including whole lung (WL) and PTV contours were taken from the original clinical plans. IMRT with five to eight coplanar 6‑MV flat beams was used for the patient reported in this paper. The beam directions were customized for individual patients. Intensity modulation was performed using the direct machine parameter optimization (DMPO) algorithm, where the maximum number of multileaf collimator (MLC) segments was set at ten. For each plan, dose distributions were calculated using the collapsed cone convolution algorithm (CCC) with a calculation grid of 3 mm.

The lung-avoidance plan is designed to maximize the protection of functional and radiosensitive lungs. For this purpose, five levels of lung substructures were generated: intersection of high-ventilation and high-density areas (level 1), union of high-ventilation and high-density areas (level 2), intersection of medium-ventilation and medium-density areas (level 3), union of medium-ventilation and medium-density areas (level 4), and the remaining lung regions (level 5). The level 4 and 5 substructures were unconstrained during optimization. Level 3 was assigned the same objectives as the clinical plan, while gradually stricter objectives were set for levels 2 and 1. In the lung-avoidance plan, the anatomical objectives for the lung were replaced by functional objectives such as level 1 (detailed optimization objectives and weights are listed in Supplementary Appendix Table A2).

### Data analysis

In the case of lung-avoidance radiotherapy, evaluating dose–volume parameters alone is insufficient; rather, an assessment is needed that combines both dose and function. We used standard dose–volume and dose–function metrics. Standard dose metrics included the D_mean_, D_2_, D_98_, of the PTV, conformity index (CI), homogeneity index (HI; defined as D_5_/D_95_), maximum dose of spinal cord, and V30Gy, V40Gy, and mean dose of heart (MHD), V5Gy, V20Gy, V30Gy, and mean lung dose (MLD) of the whole lung.

The dose–volume parameters in the ventilation as well as density zones were obtained in a fashion similar to the clinical dosimetric metrics V5Gy, V20Gy, V30Gy, and mean lung dose (MLD). The cumulative lung volume that received higher than 20 Gy in the high-ventilation region was obtained and normalized to the total healthy lung volume to derive the vV20_high. The vV5_high for the volume fraction that received higher than 5 Gy, vV30_high for the fraction that received higher than 30 Gy, and vMLD_high for MLD in the high-ventilation region were also derived in this manner. This process was repeated to obtain analogous dosimetric parameters in the high-density region (dV20_high, dV5_high, dV30_high, and dMLD_high).

In addition, the Dice coefficient was adopted to measure the relationship between ventilation and density lungs,

3 $$Dice\,\textit{coefficient}=\frac{2| V_{A}\cap V_{B}| }{\left| V_{A}\right| +| V_{B}| }$$

where V_A_ represents the volume of ventilation lung and V_B_ represents the density lung.

### Statistics

Nonparametric statistical tests were used. Wilcoxon rank-sum test was used as a paired test of the significance of differences in clinical and lung-avoidance plans within 20 patients. *P*-values of less than 0.05 were considered statistically significant.

## Results

### Clinical and lung-avoidance plans for an example patient

**Fig. 2:**
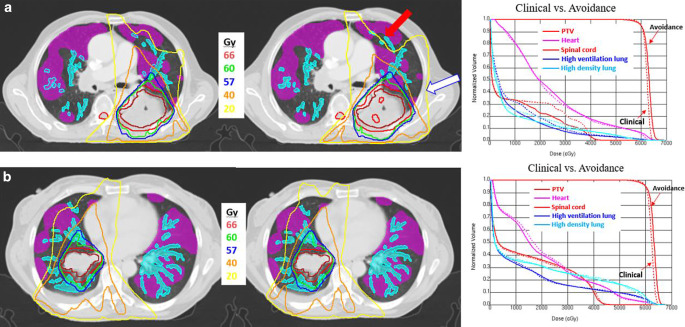
Example isodose distributions and dose–volume histograms (DVHs) of clinical and lung-avoidance plans of two patients. In isodose distributions, high-ventilation lung region is shaded *rose red*, high-density lung region is shaded *aqua green*; GTV: *scarlet*; PTV: *red*; heart: *rose red*, and spinal cord: *red*. The *red arrows* highlight where the lung-avoidance plan was able to spare functional and high-density portions of the lung, while the *white arrows* demonstrate how the lung-avoidance plan deposited higher doses to nonfunctional/low-density lung (when compared with the clinical plan). In DVHs, clinical plans are represented by *solid curves*, and lung-avoidance plans by *dashed lines*

Fig. 2 shows the isodose distributions and dose–volume histograms (DVHs) of the clinical and lung-avoidance plans for two patients. The arrow in the patient’s dose distribution shows that the lung-avoidance plan can protect high-ventilation and high-density lung areas, depositing doses in medium- or low-function/density areas. As shown in Fig. 2a, highly significant reductions could be obtained with lung-avoidance planning for high-ventilation/high-density V5 and V20. However, there were no noticeable differences in the dose distribution and DVH of patient b (Fig. 2b). This may be because the H‑V and H‑D areas are much more complex and scattered around the target area and it was nearly impossible to avoid dosing in these areas.

### Dice coefficient

The mean lung volume of the 20 patients was level 1, 220.92 ± 127.26 cc; level 2, 1289.48 ± 329.18 cc; level 3, 330.57 ± 109.60 cc; level 4, 572.08 ± 241.14 cc; and level 5, 483.97 ± 216.00 cc.

The Dice coefficient of high-ventilation and high-density lungs was 0.25 ± 0.15 (range: 0.09–0.69), and that of medium-ventilation and medium-density lungs was 0.28 ± 0.05 (range: 0.15–0.41).

### Comparison of total lung metrics between clinical and lung-avoidance plans

**Fig. 3:**
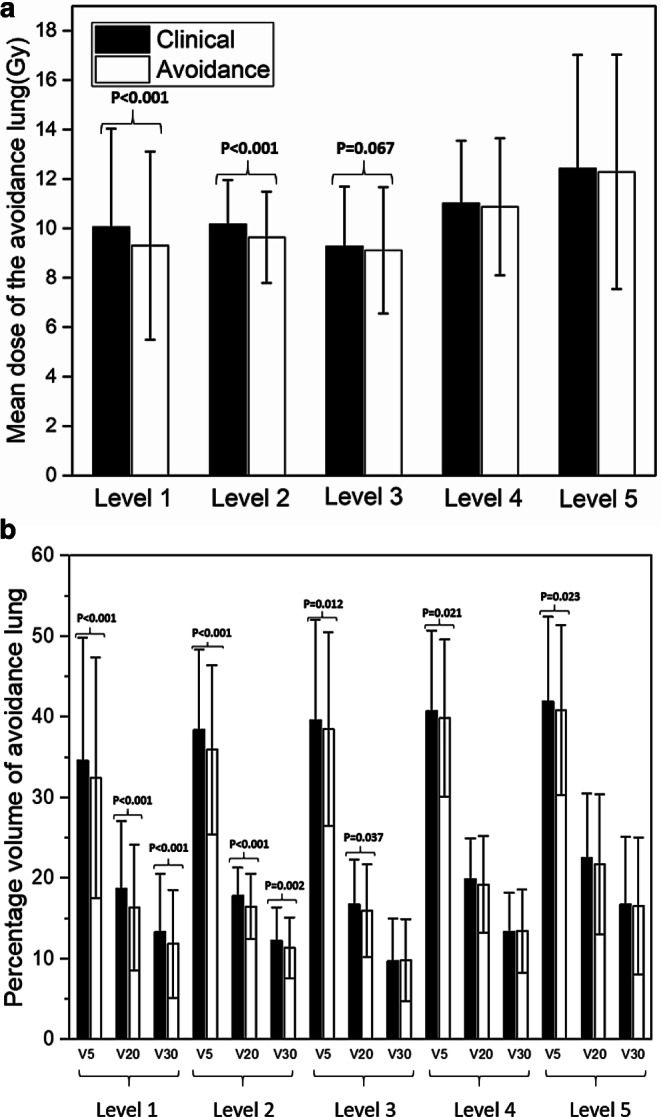
Comparisons of **a** mean dose (mean ± standard deviation) and **b** percentage of volume receiving ≥ 5, ≥ 20, ≥ 30 Gy (*V5*, *V20*, *V30*) for the five kinds of lung regions between clinical and lung-avoidance treatment plans for 20 lung cancer patients. The *p*-values are shown only for statistically significant differences between metrics of clinical and lung-avoidance planning

Fig. 3 shows the comparisons of the evaluation metrics for the five lung regions (levels 1 to 5) between the clinical and lung-avoidance plans. A significant improvement with lung-avoidance planning was observed for substructures including high-ventilation and high-density lung regions (levels 1 and 2), e.g., the average MLD from 10.05 Gy to 9.30 Gy (*p* < 0.001) and the V20 from 18.71% to 16.35% (*p* < 0.001) for level 1.

The comparison of the MLD in five levels of lung regions in clinical and lung-avoidance plans is shown in Fig. 3a. The mean values in levels 1, 2, and 3 lung regions of the lung-avoidance plans were significantly lower than those in the clinical plans; the lung mean values in levels 4 and 5 were also lower, but no statistical significance was found. For the MLD, the decreases in level 1, 2, and 3 lung regions were 0.75 Gy, 0.53 Gy, and 0.16 Gy, respectively.

It can be concluded from Fig. 3 that the much stricter objectives in the lung-avoidance plan for level 1 and level 2, which involve the high-ventilation and high-density portions, resulted in significant improvements (*p* < 0.001) in the volume metrics, including the V5, V20, and V30. For level 3, the lung-avoidance plan shared the same objective with the clinical plan, acquiring a significant improvement in V5 and V20, while no significant difference was observed for V30 (*p* > 0.05). In addition, there were no objective constraints set for level 4 and 5 lung regions; therefore, no significant differences were observed for V20, V30, and MLD in level 4 and 5 lungs.

### The comparison of OARs between clinical and lung-avoidance plans

Table 2 lists the dose metrics for the whole lung, spinal cord, and heart in the clinical and lung-avoidance plans for 20 patients. The whole-lung dose indexes (V5, V20, V30, and MLD) in the lung-avoidance plan were lower than those in the clinical plan and were statistically significant (*p* < 0.05). For the spinal cord and heart, the dose index in the lung-avoidance plan was slightly higher than that in the clinical plan, but the difference was small and there was no significant difference (*p* > 0.05).

**Table 2:** Critical organ metrics of clinical and lung-avoidance plans for 20 lung cancer patients

OAR	Metric	Clinical	Lung-avoidance	*P*-value
Total lung	V5 (%)	39.92 ± 8.66	38.44 ± 8.47	0.001
	V20 (%)	19.99 ± 3.59	18.73 ± 4.03	< 0.001
	V30 (%)	13.99 ± 4.11	13.51 ± 3.86	0.017
	MLD (Gy)	11.12 ± 1.79	10.75 ± 1.87	< 0.001
Spinal cord	D_max_ (Gy)	34.99 ± 13.85	35.33 ± 14.90	0.108
Heart	V30 (%)	29.32 ± 26.14	29.37 ± 23.57	0.191
	V40 (%)	16.63 ± 12.07	17.03 ± 11.30	0.156
	MHD (Gy)	18.36 ± 9.18	18.58 ± 9.69	0.332

### Comparison of PTV metrics

The PTV indicators for the two planning strategies are listed in Table 3. Compared with those in the clinical plan, the mean dose and D_2_ of PTV in the lung-avoidance plan increased, while D_98_ decreased, and the differences were statistically significant. In addition, CI decreased from 0.72 ± 0.10 to 0.66 ± 0.08 (*P* < 0.001), with a relative decrease of 8.33%, and an increase of 1.87% occurred in HI, from 1.07 ± 0.01 to 1.09 ± 0.01 (*P* < 0.001). Monitor units of the lung-avoidance plan were higher than those of the clinical plan (502 ± 126 vs. 512 ± 122), and the number of beam segments was also increased (28 ± 8 vs. 29 ± 8).

**Table 3:** PTV metrics of clinical and lung-avoidance treatment plans for 20 lung cancer patients

Metric	Clinical	Lung-avoidance	*P*-value
Mean Dose (Gy)	60.16 ± 3.77	60.75 ± 3.75	< 0.001
D_2_ (Gy)	62.29 ± 3.86	63.36 ± 3.89	< 0.001
D_98_ (Gy)	56.73 ± 3.75	56.19 ± 3.80	< 0.001
Conformity index	0.72 ± 0.10	0.66 ± 0.08	< 0.001
Homogeneity index	1.07 ± 0.01	1.09 ± 0.01	< 0.001
Monitor units	502 ± 126	512 ± 122	0.573
Number of segments	28 ± 8	29 ± 8	0.447

### Dose metric comparisons for the high-ventilation and high-density lung volume

Table 4 shows the comparison of volume indexes and mean values for the high-ventilation and high-density lung regions. Compared with the clinical plan, the average decrease in V5 in the H‑V region was 2.07% and was 2.16% in the H‑D region for the lung-avoidance plan; the V20 decreased by 1.82% (H-V) and 2.16% (H-D), and the MLD decreased by 0.59 Gy (H-V) and 0.57 Gy (H-D), respectively. The differences in the above indexes were all statistically significant.

**Table 4:** High-ventilation and high-density volume metrics of clinical and lung-avoidance treatment plans for 20 lung cancer patients

Metric	CL	AV	Relative difference (range)	Absolute difference (range)	*P*-value
vV5_high (%)	36.70 ± 10.44	34.63 ± 10.27	5.64 (−0.76–13.85)	2.07 (−0.30–3.96)	< 0.001
vV20_high (%)	16.92 ± 4.00	15.10 ± 4.03	10.77 (3.94–24.68)	1.82 (0.59–4.60)	< 0.001
vV30_high (%)	11.24 ± 4.31	10.35 ± 3.92	7.86 (−16.37–21.06)	0.88 (−0.98–1.93)	0.001
vMLD_high (Gy)	9.58 ± 1.93	9.00 ± 1.90	6.13 (3.31–15.49)	0.59 (0.317–1.55)	< 0.001
dV5_high (%)	58.71 ± 100.94	56.55 ± 98.56	3.68 (−1.80–18.17)	2.16 (−0.49–12.27)	< 0.001
dV20_high (%)	27.81 ± 32.47	25.65 ± 31.11	7.78 (−11.50–22.38)	2.16 (−0.46–8.26)	< 0.001
dV30_high (%)	19.94 ± 21.95	18.84 ± 22.57	5.51 (−1.74–27.16)	1.10 (−1.88–2.82)	0.001
dMLD_high (Gy)	10.94 ± 4.07	10.37 ± 4.08	5.19 (1.17–18.14)	0.57 (0.193–1.143)	< 0.001

*CL* clinical plan; *AV* lung-avoidance plan

Except for patients 19 and 11, the V5, V20, V30, and MLD in the high-ventilation and high-density lung areas were all decreased in the other 18 patients. Even though the V5 of patient 19 increased by 0.3% (vV5_high) and 0.49% (dV5_high), the other indicators for this patient were still lower than those of the clinical plan (vMLD_high decreased 0.84 Gy and dMLD_high decreased 1.08 Gy). The same situation was observed for patient 11.

## Discussion

In this study, we proposed a radiotherapy planning strategy sparing the high-function and high-sensitivity lung at the same time. The strategy merged 4DCT ventilation imaging and CT characteristics, segmenting high-ventilation and high-density lung substructures. After the process of intersection and union of two kinds of characteristic lung portions, five levels of lung regions were generated. Different optimization objectives were set to five lung regions to protect specific lung portions. The results showed that the MLD, V5, V20, and V30 of the high-ventilation and high-density substructures were significantly decreased for the lung-avoidance plan compared to those of the clinical plan, while the OAR metrics were comparable. The lung-avoidance plan can provide obvious clinical benefits, reducing lung toxicity and improving quality of life. This study provided a brand-new attempt at functional imaging combined with conventional CT to guide RT, and collected important data for the development of functional lung clinical trials. Further studies, including clinical trials, are needed to determine its clinical significance.

Other scholars have carried out previous studies on functional avoidance plans for NSCLC. For instance, Yaremko [17] carried out a double-blinded clinical trial of functional lung-avoidance RT based on ^3^He MRI. These studies indicated that functional lung avoidance is a possible development direction for RT [4, 5, 18–20]. On the basis of former research findings, we added the design of a lung-avoidance plan for high-density lungs, which may reflect the dynamic changes in heterogeneous lungs during RT. The ΔHU after radiotherapy is dependent on ΔHU_max_ and dose [15]. Since ΔHU_max_ was found to be linearly dependent on the baseline HU (HU_0_) of lung portions [21], this means that the ΔHU of the lung after radiotherapy is linearly related to the baseline density. ΔHU may be a substitute for lung injury [14, 22, 23], avoiding high-density areas can spare healthy lungs, which are vulnerable to radiation damage.

During radiotherapy, as the tumor shrinks and the atelectasis area is released, the functional status of the lung portions tends to change, which results in a reduction in the clinical benefits of the functional lung avoidance plan. In this study, we attempted to avoid high-density lung substructures while avoiding functional areas. The change in the value of HU in high-density areas during radiotherapy is greater than that in low-density areas. To a certain extent, can we assume that the probability of functional changes in high-density areas is higher and can make up for the changes in lung function states in the implementation of functional avoidance radiotherapy. However, this is only a hypothesis. The correlation between density and functional changes still needs further research.

This study showed that the Dice coefficients of high-ventilation and high-density areas of 20 patients were not high, which confirmed that the coincidence degree between the two characteristic lung regions was small, and there was no strong correlation between high-function lung and the baseline density value. The comparison between clinical and lung-avoidance plans showed that the lung-avoidance plan could significantly improve V5, V20, V30, and MLD in level 1 and 2 lung regions and V5, V20, and MLD in level 3 areas. Although we did not assign objectives for level 4 and 5 lung regions, the V5 values in levels 4 and 5 were also lower than those of the clinical plan and were statistically significant. This is because the distributions of the five lung regions are overlapping, which will lessen a certain degree of dose to the low-density and low-ventilation areas adjacent to levels 1–3 during reverse planning, especially impacting the low-dose area.

Table 2 showed that compared to the clinical plan, the dose of OARs except for the whole lung had a certain degree of increase in the lung-avoidance plan, but there was no significant difference. In addition, D_mean_, D_2_, and D_98_ in the lung-avoidance plan worsened, CI and HI also became worse, and the MU and number of segments increased slightly. Among those PTV metrics, only the MU and the number of segments were not statistically significant. As expected, the dose limitation for different substructures in the lung-avoidance plan increases the complexity of the plan, and the protection of high-ventilation and high-density lungs comes at the cost of sacrificing CI and HI to some extent.

Among the 20 patients in this study, two patients had a slight increase in the V5 or V30 compared to those in the clinical plan under the optimized settings of the lung-avoidance plan. The distributions of the high-ventilation and high-density areas of these two patients are similar to Fig. 2b, which were close to the PTV and distributed scattered. This may be the reason why the optimization process takes more time to adjust, but the dose is still not decreased. Although the value of V5 or V30 is not ideal, the other indexes of these two patients are still decreased, especially MLD (decreased from 9 Gy to 8.77 Gy and 12.38 Gy to 11.60 Gy, respectively), which can also indicate that their high-ventilation and high-density lungs were spared to a certain degree.

According to our data, compared to the clinical plan, the lung-avoidance plan required 55 ± 12 additional minutes. The lung-avoidance plan required more time due to the extra process of scanning 4DCT and calculating and segmenting high-ventilation and high-density areas; moreover, the calculation time of TPS was also slightly increased.

This study encountered technical limitations related to CT ventilation imaging, including 4DCT image artifacts, which reduce the accuracy and precision of CT ventilation [24, 25]. Future strategies to improve 4DCT will also improve CT ventilation imaging. In addition, the theoretical premise that ΔHU is a substitute for lung injury is controversial. For example, the airway and blood vessels outside the high-density area also need additional protection, which is also a challenge for all lung-avoidance plans. At present, there is no clinical application of lung-area-avoidance plans based on two kinds of characteristics, and our research provides only ideas and references for future clinical applications.

## Conclusion

This study attempted to combine 4DCT ventilation images and conventional CT images to design a lung-avoidance radiotherapy planning strategy that can protect high-ventilation and high-density lung regions at the same time. We divided the whole lung into five levels and set different optimization objectives. With spinal cord and heart doses comparable to clinical plans, the lung-avoidance plans achieved a significant improvement in dose metrics for high-function and high-density lung areas. This study demonstrated the potential clinical benefits of functional imaging combined with conventional CT-guided radiotherapy, which can reduce pulmonary toxicity and improve quality of life. Further research, including clinical trials, is needed to determine its clinical significance.

## Supplementary Information

Supplementary Appendix: Planning dose constraints and optimization objectives.
