# Supplementary material for: A novel lung-avoidance planning strategy based on 4DCT ventilation imaging and CT density characteristics for stage III non-small-cell lung cancer patients
Source: Strahlenther Onkol. 2021 Aug 5;197(12):1084–92. doi: 10.1007/s00066-021-01821-1 (PMC8604857; doi:10.1007/s00066-021-01821-1)
Supplement: Supplementary file 1 — Supplementary Appendix: Planning dose constraints and optimization objectives. [file 66_2021_1821_MOESM1_ESM.docx]

Table A1 Planning dose constraints that should be achieved for a clinically acceptable plan.

| Structure | Objectives | Expected dose |
| --- | --- | --- |
| PTV | V_100%_ | >95% |
|  | V_95%_ | >99% |
|  | Dmax | <115% of prescribed dose |
| Total Lung | MLD | 17Gy |
|  | V20 | <25% |
|  | V5 | <60% |
| Heart | MHD | 26Gy |
|  | V30 | <40% |
|  | V40 | <30% |
| Spinal Cord | D_max_ | <50Gy |

Abbreviations: PTV, planning tumor volume. D_max_, maximal dose. MHD, mean heart dose. MLD, mean lung dose.

Table A2 Starting optimization objectives and weights applied during clinical plan optimization (CL) and lung-avoidance plan optimization (AV). In case the objective is used in both planning techniques, it is labeled as ALL. If the objective is used merely in clinical plan or lung-avoidance plan, it is labeled as CL or AV, respectively.

| Structure | Planning (CL, AV or ALL) | Type | Dose(cGy) | Volume (%) | Priority | Comprise (Yes or No) |
| --- | --- | --- | --- | --- | --- | --- |
| Total Lung | CL | Max DVH | 500 | 40 | medium | Yes |
|  | CL | Max DVH | 2000 | 20 | medium | Yes |
|  | CL | Max DVH | 3000 | 15 | medium | Yes |
| Spinal Cord+2mm expansion | ALL | Max dose | 4400 |  | High | No |
| Spinal Cord+5mm expansion | ALL | Max dose | 4500 |  | High | No |
| Heart | ALL | Max DVH | 3000 | 25 | High | Yes |
|  | ALL | Max DVH | 4000 | 35 | High | Yes |
| Level 1 | AV | Max DVH | 500 | 30 | High | Yes |
|  | AV | Max DVH | 2000 | 18 | High | Yes |
|  | AV | Max DVH | 3000 | 10 | High | Yes |
| Level 2 | AV | Max DVH | 500 | 35 | High | Yes |
|  | AV | Max DVH | 2000 | 21 | High | Yes |
|  | AV | Max DVH | 3000 | 12 | High | Yes |
| Level 3 | AV | Max DVH | 500 | 40 | medium | Yes |
|  | AV | Max DVH | 2000 | 20 | medium | Yes |
|  | AV | Max DVH | 3000 | 15 | medium | Yes |
| Level 4 & Level 5 | ALL | None |  |  |  |  |
